# Global epidemiology of vaccine-associated intussusception in children and adolescents, 1968 to 2024: An international pharmacovigilance study

**DOI:** 10.1097/MD.0000000000045878

**Published:** 2025-11-21

**Authors:** Tae Hyeong Kim, Hyesu Jo, Damiano Pizzol, Lee Smith, Dong Keon Yon

**Affiliations:** a Department of Pediatrics, Kyung Hee University Hospital at Gangdong, Kyung Hee University College of Medicine, Seoul, South Korea; b Center for Digital Health, Medical Science Research Institute, Kyung Hee University College of Medicine, Seoul, South Korea; c Department of Regulatory Science, Kyung Hee University, Seoul, South Korea; d Health Unit Eni, Maputo, Mozambique; e Health Unit, Eni, San Donato Milanese, Italy; f Centre for Health, Performance and Wellbeing, Anglia Ruskin University, Cambridge, UK; g Department of Public Health, Faculty of Medicine, Biruni University, Istanbul, Turkey; h Department of Pediatrics, Kyung Hee University College of Medicine, Seoul, South Korea.

**Keywords:** epidemiology, vaccine, vaccine-associated intussusception

## Abstract

Rotavirus vaccine associated intussusception has been investigated to some extent, limited research exists on intussusception caused by all vaccines from a comprehensive, global perspective. Thus, this study aimed to investigate the global burden of vaccine-associated intussusception using a comprehensive pharmacovigilance database. This study was conducted using data from the global pharmacovigilance database from 1968 to 2024. Reports of intussusception were identified by MedDRA code and categorized by vaccine type, age, sex, and region. Statistical disproportionality analysis was performed using reporting odds ratios (ROR) with 95% confidence interval (CI) and information components (IC) with IC_025_ to assess vaccine-specific signal detections. Among 35 million vaccine-associated total adverse events, 12,055 reports of vaccine-associated intussusception were identified. Rotavirus vaccines exhibited the strongest signal detections with intussusception (ROR, 70.16 [95% CI, 67.56–72.86]; IC, 5.27 [IC_025_, 5.22]), followed by diphtheria, tetanus, acellular pertussis, polio, and Haemophilus influenzae type b (ROR, 3.82 [95% CI, 3.67–3.97]; IC, 1.55 [IC_025_, 1.49]) and pneumococcal vaccines (ROR, 8.54 [95% CI, 8.13–8.96]; IC, 2.84 [IC_025_, 2.77]). The mean time to onset was 2.77 days, with a low fatality rate (0.07%). While our findings do not allow for causal conclusions, the results of this study suggest that rotavirus vaccines may be associated with a slight increase in the risk of intussusception. However, it is important to note that their benefits in preventing severe diarrheal disease far outweigh the risks.

## 1. Introduction

Mortality from intussusception in children is rare, but the condition remains a leading cause of abdominal emergencies in children.^[1]^ Notably, since the introduction of the rotavirus vaccine in the 2000s, a slight increase in the risk of intussusception has been observed, with some evidence suggesting a signal detections between the vaccine and the condition.^[2]^ However, despite these findings, research on intussusception linked to other vaccines remains limited.

Vaccines have played a pivotal role in reducing the global burden of infectious diseases, significantly reducing mortality and morbidity. Despite their efficacy and safety in most cases, adverse reactions have been rarely reported,^[3–5]^ including gastrointestinal complications such as intussusception.^[4,6,7]^ Intussusception, a condition in which one segment of the intestine enters into an adjacent segment, is especially significant in pediatric populations. Among vaccines, rotavirus vaccines have been frequently associated with intussusception, raising important discussions for the risk-benefit balance and safety profile.

Previous studies on intussusception have primarily focused on specific vaccines or small population groups, limiting our understanding of the global burden of vaccine-associated intussusception and the characteristics of populations at risk.^[4,6]^ To address these gaps, comprehensive analyses of vaccine-associated intussusception using data from robust databases are needed. Given this background, this study aims to assess the worldwide burden of vaccine-associated intussusception, identify populations at increased age- and gender-specific risk, and evaluate signal detections between different vaccines and intussusception through statistical disproportionality analysis using the World Health Organization (WHO) pharmacovigilance database.

## 2. Methods

### 2.1. Data source

This study utilized data from VigiBase, the global pharmacovigilance database, which aggregates adverse reaction reports from over 140 countries since 1968, with data included up to 2024.^[8]^ This extensive database is managed by the Uppsala Monitoring Centre under the WHO International Drug Monitoring Program. It comprises total individual case safety reports associated with suspected adverse reactions and serves as an important resource for global pharmacovigilance and drug safety analysis.^[9]^ The study protocol received ethical approval and anonymized data were used to ensure privacy and confidentiality. Approval was granted by the Institutional Review Boards of Kyung Hee University.

### 2.2. Selection of cases and definition

This study analyzed data on vaccine-associated intussusception from 1968 to 2024, categorizing vaccines into 12 distinct categories: diphtheria, tetanus, acellular pertussis, polio, and *Haemophilus influenzae* type b, meningococcal, pneumococcal, tuberculosis, influenza, hepatitis A, hepatitis B, measles, mumps, and rubella, rotavirus diarrhea, zoster, COVID-19 mRNA, and other (ad5-vectored COVID-19, anthrax, brucellosis, cholera, dengue, Ebola, encephalitis, inactivated whole-virus COVID-19, leptospirosis, papillomavirus, plague, rabies, smallpox, typhoid, typhus, and yellow fever) vaccines.

Intussusception was defined by Medical Dictionary for Regulatory Activities version 26.0, employed to compile adverse events, focusing on the preferred terms detailed in Table S1, Supplemental Digital Content, https://links.lww.com/MD/Q661. According to WHO causality assessment guidelines, only vaccines designated as “suspected” were included in the analysis of disproportional signal detection with intussusception (Table S1, Supplemental Digital Content, https://links.lww.com/MD/Q661).

### 2.3. Data collection

This study systematically analyzed reports of suspected vaccine-associated intussusception, conducting a comprehensive evaluation. The dataset was derived from individual case safety reports reported by patients, healthcare professionals, and pharmaceutical companies during the post-market surveillance period. The dataset is categorized into 3 primary domains: patient demographics (age groups: 0–27 days, 28 days–23 months, and 2–11 years; sex: male and female; and reporting regions: Africa, the Americas, Southeast Asia, Europe, Eastern Mediterranean, and Western Pacific), adverse reactions (reporting periods: 1968–1999, 2000–2004, 2005–2009, 2010–2014, 2015–2019, and 2020–2024; time to onset [TTO]; and outcomes: recovered/recovering, not recovered, fatal, death, and unknown), and drug information (vaccine type and suspected vaccine).^[10,11]^

### 2.4. Statistical analysis

Disproportionality analysis was conducted to evaluate the signal detections between specific vaccines and intussusception by comparing the frequency of intussusception reported for a particular vaccine to the frequency of reports not-reported for that vaccine across all other vaccines. This method identifies significant signal detections between vaccines and adverse events by contrasting reporting patterns. Two pharmacovigilance indicators were utilized for this analysis. The reporting odds ratio (ROR), a frequentist measure, quantifies the likelihood of intussusception occurring after specific vaccines compared to all other adverse reactions by constructing contingency tables based on reported events.^[10,11]^ The ROR lower bound of the 95% confidence interval (CI) exceeding 1.00 indicates a significant signal detection between the vaccine and intussusception. The information component (IC), derived through a Bayesian method that compares observed and expected frequencies of intussusception. An IC_025_ >0.00 was considered indicative of a statistically significant signal detection. Statistical significance was determined using a 2-sided *P*-value of <.05. All analyses were conducted with SAS software (version 9.4; SAS Institute Inc., Cary).

## 3. Results

From 1968 to 2024, a total of 11,152 reports of vaccine-associated intussusception were reported out of 2.6 million vaccine-associated adverse reactions. The majority of reports originated from the Americas, accounting for 74.32% (8288 reports) of reports, followed by the Western Pacific region with 15.12% and Europe with 9.57%. In contrast, reports were significantly underreported in Africa and Southeast Asia, suggesting potential limitations in pharmacovigilance infrastructure in these regions. A noticeable increase in intussusception reports were observed after 2010, coinciding with the global introduction of rotavirus vaccines. The highest number of reports, 37.35%, were recorded between 2020 and 2024, with elevated reporting levels persisting into the 2020s (Table 1).

**Table 1:** Baseline characteristics of reports on vaccine-associated intussusception (n = 11,152).

Variables		Number (%)
Region reporting	African region	56 (0.50)
	Region of the Americas	8288 (74.32)
	Southeast Asia region	51 (0.46)
	European Region	1067 (9.57)
	Eastern Mediterranean region	4 (0.04)
	Western Pacific region	1686 (15.12)
Reporting year	1968–1999	2 (0.02)
	2000–2004	9 (0.08)
	2005–2009	3 (0.03)
	2010–2014	21 (0.19)
	2015–2019	30 (0.27)
	2020–2024	4165 (37.35)
Reporter qualification	Health professional	2703 (24.24)
	Non-health professional	104 (0.93)
Sex	Male	6463 (57.95)
	Female	4621 (41.44)
	Unknown	68 (0.61)
Age, years	0–27 days	61 (0.55)
	28 days to 23 months	11,022 (98.83)
	2–11 years	69 (0.62)
TTO, days, mean (SD)		2.77 (27.31)
Drug class	DTaP-IPV-Hib vaccines	3608 (32.35)
	Meningococcal vaccines	47 (0.42)
	Pneumococcal vaccines	1969 (17.66)
	Tuberculosis vaccines	12 (0.11)
	Influenza vaccines	82 (0.74)
	Hepatitis A vaccines	9 (0.08)
	Hepatitis B vaccines	563 (5.05)
	MMR vaccines	41 (0.37)
	Rotavirus diarrhea vaccines	4788 (42.93)
	Zoster vaccines	18 (0.16)
	COVID-19 mRNA vaccines	7 (0.06)
	Others*	8 (0.07)
Fatal outcomes	Recovered	8466 (75.91)
	Not Recovered	187 (1.68)
	Fatal	8 (0.07)
	Death	0 (0.00)
	Unknown	2491 (22.34)
Single drug suspected		11,152 (100.00)

DTaP-IPV-Hib = diphtheria, tetanus, acellular pertussis, polio, and *Haemophilus influenzae* type b, IQR = interquartile range, MMR = measles, mumps, and rubella, SD = standard deviation, TTO = time to onset.

* Others: Ad5-vectored COVID-19, anthrax, brucellosis, cholera, dengue, Ebola, encephalitis, inactivated whole-virus COVID-19, leptospirosis, papillomavirus, plague, rabies, smallpox, typhoid, typhus, and yellow fever.

Most reports were reported in children aged 28 days to 23 months, comprising 98.83% (11,022 reports) of all reports, aligning with the typical schedule for rotavirus vaccination. Neonates younger than 28 days contributed 0.51%, while older children between 2 and 11 years of age accounted for only 0.62%. Regarding sex distribution, a slightly higher proportion of reports were observed in males (57.95%) compared to females (41.44%) (Table 1).

The mean TTO for intussusception symptoms following vaccination was just 2.77 days, indicating that symptoms typically appeared rapidly after vaccine administration. The majority of reports recovered successfully, with a recovery rate of 75.91%. Fatalities were extremely rare, with only 8 reported globally, representing just 0.07% of reports (Table 1).

**Figure 1.**
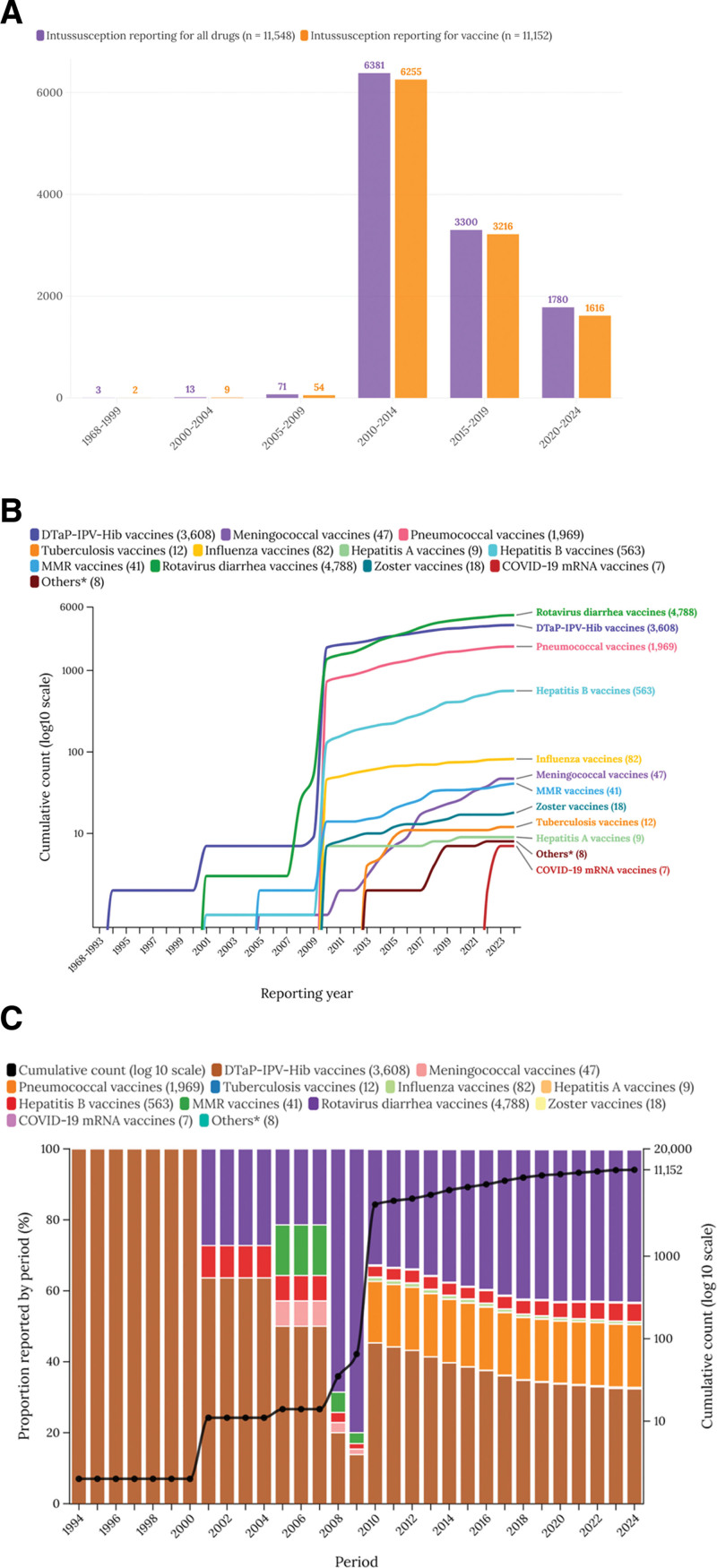
Temporal trends (A) and cumulative number of reports (B and C) of vaccine-associated intussusception by year. *Others: Ad5-vectored COVID-19, anthrax, brucellosis, cholera, dengue, Ebola, encephalitis, inactivated whole-virus COVID-19, leptospirosis, papillomavirus, plague, rabies, smallpox, typhoid, typhus, and yellow fever.

**Figure 2.**
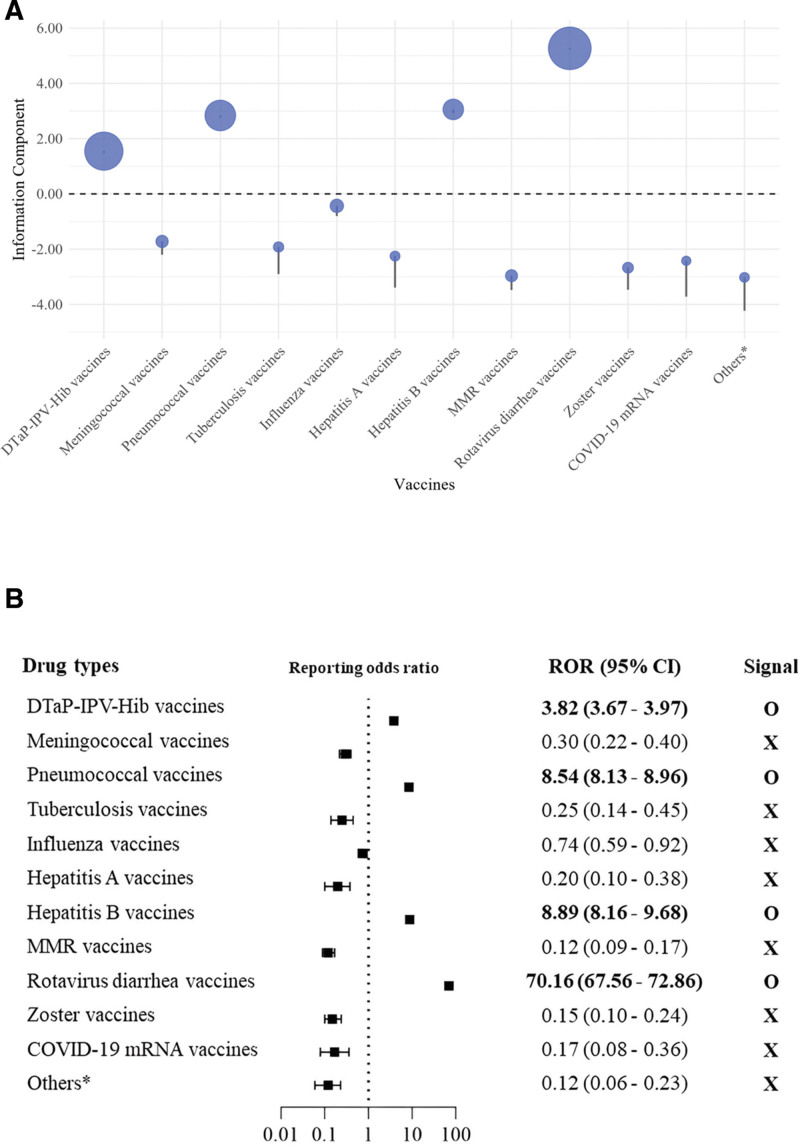
Disproportionality analysis of subgroups based on IC_025_ (A) and ROR (B) values in vaccine-associated intussusception. *Others: Ad5-vectored COVID-19, anthrax, brucellosis, cholera, dengue, Ebola, encephalitis, inactivated whole-virus COVID-19, leptospirosis, papillomavirus, plague, rabies, smallpox, typhoid, typhus, and yellow fever. ROR = reporting odds ratios.

Rotavirus vaccines were the highest associated with intussusception (ROR, 70.16 [95% CI, 67.56–72.86]; IC, 5.27 [IC_025_, 5.22]), indicating a substantial statistical signal detection. Other vaccines, such as diphtheria, tetanus, acellular pertussis, polio, and Haemophilus influenzae type b (ROR, 3.82 [95% CI, 3.67–3.97]; IC, 1.55 [IC_025_, 1.49]) and pneumococcal vaccines (ROR, 8.54 [95% CI, 8.13–8.96]; IC, 2.84 [IC_025_, 2.77]), also exhibited significant signal detection, though to a lesser extent than rotavirus vaccines. Conversely, COVID-19 mRNA vaccines showed no significant signal detections with intussusception, as reflected in ROR values below 1.00 and negative IC_025_ values (Table 2, Figs. 1 and 2).

**Table 2:** Disproportionality analysis of subgroups in vaccine-associated intussusception.

	Total	Vaccine-associated intussusception			IC (IC_025_) based on age		
		Observed	ROR (95% CI)	IC (IC_025_)	0 to 27 days	28 days to 23 months	2 to 11 years
Total	1,534,983	11,152	**94.45 (85.43–104.42**)	**2.07 (2.04**)	**3.17 (2.74**)	**1.58 (1.55**)	**1.28 (0.88**)
Sex difference							
Male	794,331	6463	**95.06 (83.59–108.10**)	**2.12 (2.08**)	**3.16 (2.59**)	**1.60 (1.56**)	**1.10 (0.54**)
Female	708,795	4621	**95.00 (80.91–111.55**)	**2.00 (1.96**)	**2.78 (2.13**)	**1.55 (1.50**)	**1.37 (0.76**)
Vaccine types							
DTaP-IPV-Hib vaccines	714,035	3608	**3.82 (3.67–3.97**)	**1.55 (1.49**)	**2.21 (1.23**)	**0.95 (0.90**)	−0.03 (−1.05)
Meningococcal vaccines	90,236	47	0.30 (0.22–0.40)	−1.72 (−2.20)	1.07 (−2.71)	−2.18 (−2.67)	−0.64 (−4.42)
Pneumococcal vaccines	159,037	1969	**8.54 (8.13–8.96**)	**2.84 (2.77**)	**3.24 (2.21**)	**1.96 (1.89**)	1.05 (−1.02)
Tuberculosis vaccines	27,193	12	025 (0.14–0.45)	−1.92 (−2.90)	N/A	−2.85 (−3.87)	1.30 (−2.49)
Influenza vaccines	64,138	82	0.74 (0.59–0.92)	−0.43 (−0.80)	N/A	0.18 (−0.22)	**2.07 (1.10**)
Hepatitis A vaccines	25,968	9	0.20 (0.10–0.38)	−2.25 (−3.39)	N/A	−2.47 (−3.69)	0.31 (−3.48)
Hepatitis B vaccines	38,851	563	**8.89 (8.16–9.68**)	**3.06 (2.92**)	**2.19 (0.42**)	**2.57 (2.43**)	0.39 (−3.40)
MMR vaccines	187,424	41	0.12 (0.09–0.17)	−2.96 (−3.48)	N/A	−3.51 (−4.07)	0.47 (−0.95)
Rotavirus diarrhea vaccines	71,559	4788	**70.16 (67.56–72.86**)	**5.27 (5.22**)	**5.15 (4.57**)	**4.20 (4.16**)	**5.26 (4.55**)
Zoster vaccines	68,177	18	0.15 (0.10–0.24)	−2.67 (−3.47)	N/A	−2.90 (−3.72)	−0.59 (−4.38)
COVID-19 mRNA vaccines	48,645	7	0.17 (0.08–0.36)	−2.42 (−3.72)	N/A	N/A	**1.43 (0.13**)
Others*	39,720	8	0.12 (0.06–0.23)	−3.02 (−4.23)	N/A	−2.35 (−3.91)	0.71 (−1.36)

Bold style indicates when the value of IC_025_ is >0.00 or the lower end of the ROR 95% CI is >1.00. This means it is statistically significant.

CI = confidence interval, DTaP-IPV-Hib = diphtheria, tetanus, acellular pertussis, polio, and *Haemophilus influenzae* type b, IC = information component, MMR = measles, mumps, and rubella, ROR = reporting odds ratio.

* Others: Ad5-vectored COVID-19, anthrax, brucellosis, cholera, dengue, Ebola, encephalitis, inactivated whole-virus COVID-19, leptospirosis, papillomavirus, plague, rabies, smallpox, typhoid, typhus, and yellow fever.

The analysis of concomitant adverse events indicated that adverse reactions varied significantly depending on the vaccine class. Abdominal complications were the most frequent adverse event associated with hepatitis A vaccines, occurring in 66.7% of reports, and were also common with rotavirus vaccines at 27.5%. Neurologic complications were notably prevalent with COVID-19 mRNA vaccines, accounting for 57.1% of adverse events. Psychiatric symptoms were observed in 33.3% of reports involving hepatitis A vaccines and in 20.7% of influenza vaccine reports. While dermatologic reactions were generally infrequent across all vaccines, they occurred more frequently with COVID-19 mRNA and hepatitis A vaccines, each reporting a 14.3% and 11.1%. Severe cardiac events such as arrhythmia were rare overall but were disproportionately reported with COVID-19 mRNA vaccines, affecting 57.1% of the reports associated with this vaccine class (Table 3).

**Table 3:** Vaccine class-based description of adverse reactions.

	DTaP-IPV-Hib vaccines	Meningococcal vaccines	Pneumococcal vaccines	Tuberculosis vaccines	Influenza vaccines	Hepatitis A vaccines	Hepatitis B vaccines	MMR vaccines	Rotavirus diarrhea vaccines	Zoster vaccines	COVID-19 mRNA vaccines	Others*
N observed	3608	47	1969	12	82	9	563	41	4788	18	7	8
Age, yr												
0 to 27 days	12 (0.3)	1 (2.1)	11 (0.6)	0 (0.0)	0 (0.0)	0 (0.0)	4 (0.7)	0 (0.0)	33 (0.7)	0 (0.0)	0 (0.0)	0 (0.0)
28 days to 23months	3585 (99.4)	45 (95.7)	1955 (99.3)	11 (91.7)	70 (85.4)	8 (88.9)	558 (99.1)	35 (85.4)	4733 (98.9)	17 (94.4)	0 (0.0)	5 (62.5)
2 to 11years	11 (0.3)	1 (2.1)	3 (0.2)	1 (8.3)	12 (14.6)	1 (11.1)	1 (0.2)	6 (14.6)	22 (0.5)	1 (5.6)	7 (100.0)	3 (37.5)
Sex												
Male	2112 (58.5)	28 (59.6)	1147 (58.3)	5 (41.7)	55 (67.1)	3 (33.3)	305 (54.2)	25 (61.0)	2762 (57.7)	13 (72.2)	1 (14.3)	7 (87.5)
Female	1492 (41.4)	16 (34.0)	819 (41.6)	7 (58.3)	26 (31.7)	6 (66.7)	258 (45.8)	16 (39.0)	1969 (41.1)	5 (27.8)	6 (85.7)	1 (12.5)
*TTO, days, mean (SD*)	1.5 (15.0)	17.9 (110.9)	1.4 (11.8)	3.4 (8.4)	1.0 (0.0)	1.0 (0.0)	1.2 (2.8)	1.7 (4.5)	4.4 (37.4)	1.0 (0.0)	1.0 (0.0)	1.0 (0.0)
Fatal outcomes												
Recovered	2821 (78.2)	29 (61.7)	1479 (75.1)	10 (83.3)	61 (74.4)	6 (66.7)	400 (71.0)	32 (78.0)	3606 (75.3)	14 (77.8)	1 (14.3)	7 (87.5)
Not recovered	24 (0.7)	4 (8.5)	19 (1.0)	0 (0.0)	0 (0.0)	0 (0.0)	3 (0.5)	2 (4.9)	133 (2.8)	0 (0.0)	1 (14.3)	1 (12.5)
Fatal	1 (0.0)	0 (0.0)	1 (0.1)	0 (0.0)	0 (0.0)	0 (0.0)	0 (0.0)	0 (0.0)	6 (0.1)	0 (0.0)	0 (0.0)	0 (0.0)
Death	0 (0.0)	0 (0.0)	0 (0.0)	0 (0.0)	0 (0.0)	0 (0.0)	0 (0.0)	0 (0.0)	0 (0.0)	0 (0.0)	0 (0.0)	0 (0.0)
Concomitant adverse events, %												
Coronary	0.0	0.0	0.0	0.0	0.0	0.0	0.0	0.0	0.0	0.0	0.0	0.0
Arrhythmia	1.6	4.3	2.7	0.0	0.0	0.0	2.1	4.9	2.3	0.0	57.1	0.0
Heart failure	0.7	0.0	1.1	0.0	1.2	0.0	1.2	0.0	0.7	0.0	0.0	0.0
Other cardiac diseases	0.4	0.0	0.8	0.0	0.0	0.0	0.7	0.0	0.9	0.0	0.0	0.0
Hyperthermia	5.9	10.6	10.3	25.0	23.2	11.1	8.2	14.6	7.6	5.6	0.0	0.0
Eosinophilia	0.3	0.0	0.4	8.3	0.0	11.1	1.1	0.0	0.3	0.0	0.0	0.0
Thrombocytopenia and leucopenia	1.1	0.0	1.7	8.3	2.4	0.0	2.1	2.4	1.3	0.0	14.3	0.0
Pulmonary	1.5	0.0	2.3	0.0	3.7	0.0	2.7	7.3	1.7	0.0	0.0	0.0
Infections	4.0	0.0	6.5	8.3	11.0	22.2	6.7	9.8	4.8	11.1	14.3	0.0
Abdominal	21.5	29.8	34.6	25.0	54.9	66.7	27.9	34.1	27.8	50.0	14.3	0.0
Hepato-biliary	1.2	0.0	2.1	0.0	4.9	11.1	1.8	0.0	1.3	0.0	14.3	0.0
Renal	5.6	0.0	9.6	8.3	9.8	0.0	6.2	4.9	6.7	5.6	14.3	0.0
Endocrine	0.9	0.0	1.4	0.0	1.2	0.0	0.7	2.4	1.2	0.0	0.0	0.0
Muscular	0.4	2.1	0.8	0.0	0.0	0.0	0.2	2.4	0.5	0.0	14.3	0.0
Neurologic	16.3	12.8	27.2	16.7	29.3	33.3	21.3	22.0	18.3	38.9	57.1	0.0
Psychiatric	10.8	14.9	18.4	8.3	20.7	33.3	16.9	9.8	13.8	11.1	0.0	0.0
Osteoarticular and rheumatologic	0.1	0.0	0.1	0.0	2.4	0.0	0.4	0.0	0.1	5.6	14.3	0.0
Dermatologic	0.9	2.1	1.5	0.0	4.9	11.1	1.8	4.9	1.1	0.0	14.3	0.0
Anaphylaxis	0.1	0.0	0.1	0.0	0.0	0.0	0.2	0.0	0.1	0.0	0.0	0.0
Ophthalmology	0.1	2.1	0.1	0.0	0.0	0.0	0.2	0.0	0.0	0.0	0.0	0.0

DTaP-IPV-Hib = diphtheria, tetanus, acellular pertussis, polio, and *Haemophilus influenzae* type b, MMR = measles, mumps, and rubella, SD = standard deviation, TTO = time to onset.

* Others: Ad5-vectored COVID-19, anthrax, brucellosis, cholera, dengue, Ebola, encephalitis, inactivated whole-virus COVID-19, leptospirosis, papillomavirus, plague, rabies, smallpox, typhoid, typhus, and yellow fever.

## 4. Discussion

### 4.1. Key findings

We investigated the global burden, signal detections, and characteristics of vaccine-associated intussusception through a global pharmacovigilance database. First, we identified that various vaccines were associated with intussusception, particularly rotavirus, hepatitis B, pneumococcal, and DTP-IPV-Hib vaccines. Secondly, most vaccines were found to increase the signal detection with younger individuals. Thirdly, vaccine-associated intussusception has increased since 2010, this trend is likely attributable to heightened awareness in clinical settings and the introduction of rotavirus vaccines. Additionally, the mean TTO for vaccine-associated intussusception was observed to be 2.77 days, with a fatality rate of 0.07%. Our findings suggest that clinicians, patients, and policymakers should be aware of the increasing global attention to and clinical significance of vaccine-associated intussusception.

### 4.2. Plausible underlying mechanisms

Pediatric intussusception is the most common abdominal emergency in early childhood,^[12]^ primarily affecting children aged 6 to 18 months, with a male predominance, and its incidence declines significantly after age 2. While the majority of reports are idiopathic, approximately 10% have an identifiable cause,^[13]^ including anatomical abnormalities (Meckel diverticulum, intestinal duplication), viral infections leading to mesenteric lymphadenopathy (adenovirus, rotavirus), and conditions such as Celiac disease, Crohn disease, or Henoch-Schönlein purpura.^[14–16]^ Intussusception may result from invagination at the ileocecal valve due to developmental features like reduced rigidity of the cecum or hypertrophic Peyer patches. Rarely, neoplasms like lymphoma or conditions such as malrotation can act as lead points. Functional bowel disorders and neuroenteric abnormalities, including altered peristalsis or aperistaltic segments, may also contribute.^[13]^

Interactions with the gut microbiota may further contribute to this timing.^[14–16]^ The rotavirus vaccine has been shown to alter gut microbial composition, such as shifts in Firmicutes and Bacteroidetes ratios, which can affect motility and inflammatory responses. These changes may require 24 to 48 hours to manifest and significantly impact the gut environment. Moreover, the mechanical or structural changes in the intestine, such as localized swelling or altered peristalsis, likely take time to develop and become clinically apparent.

Although biological factors are primary, delayed symptom recognition or reporting may also contribute to the observed timing,^[17]^ as some cases occurring earlier might not be detected until symptoms progress. These findings align with prior studies that indicate the highest risk of intussusception occurs within the first 3 to 7 days after vaccination, with a significant cluster around the second day.

Another study found that a Th2/Th1 cytokine imbalance was associated with a higher incidence of recurrent intussusception in children.^[18]^ Th2 cells, which are crucial for intestinal smooth muscle contractions, secrete cytokines like interleukin (IL)-4, IL-6, and IL-13, while Th1 cells secrete interferon-γ and IL-2 to regulate immunity.^[18]^ Patients with recurrent intussusception showed elevated Th2 levels, reduced Th1 levels, and significantly decreased Th1/Th2 ratios. This imbalance may be linked to enterovirus infections, a known cause of both intussusception and Th2/Th1 cytokine dysregulation.^[18,19]^

The mechanisms underlying intussusception associated with vaccines other than the rotavirus vaccine remain largely unknown. However, it is speculated that these vaccines may share similar mechanisms with the rotavirus vaccine, such as immune response activation and alterations in gut microbiota. Notably, administering multiple vaccines with such mechanisms, including the rotavirus vaccine, during infancy could have a synergistic effect, potentially increasing the likelihood of intussusception.^[20]^

### 4.3. Clinical and policy implications

The findings of this study provide a comprehensive and global overview of vaccine-associated intussusception, with particular focus on the relationship between rotavirus vaccines and intussusception in young children.^[21]^ Rotavirus vaccines, while essential in preventing severe diarrhea and associated mortality, showed the strongest signal detection with intussusception, primarily in infants under 2 years of age. This study emphasizes the importance of balancing the rare risks associated with vaccines against their considerable public health benefits.^[6,22,23]^ The high recovery rates seen for most vaccines are encouraging and underline the overall safety of vaccination programs. Serious outcomes and fatalities (death) were exceedingly rare, further supporting the favorable risk-benefit balance of these vaccines.^[24]^ However, the differences in adverse events among vaccine types highlight the importance of developing tailored postvaccination guidelines. These guidelines should focus on high-risk populations, such as young children and individuals with preexisting health conditions, to ensure optimal management and prevention of adverse reactions.

### 4.4. Comparison of previous study

The association between the first licensed rotavirus vaccine, RotaShield (a rhesus rotavirus tetravalent vaccine), and an increased risk of intussusception, estimated at one excess case per 10,000 vaccinated infants, led to its withdrawal from the U.S. market in 1999 due to safety concerns. Consequently, the 2 currently WHO-recommended rotavirus vaccines, RotaTeq (RV5, a pentavalent vaccine) and Rotarix (RV1, a monovalent vaccine), underwent extensive pre-licensure safety trials, demonstrating no risk comparable to RotaShield.^[25]^ However, post-marketing surveillance studies in high- and middle-income countries have confirmed a low-level increased risk of intussusception, estimated at 1 to 2 cases per 100,000 infants for both RV5 and RV1.^[22]^ These findings align with our study’s observation of a mild increase in intussusception risk following rotavirus vaccination, further underscoring the importance of ongoing safety monitoring to balance vaccine benefits and risks.

The Global Advisory Committee on Vaccine Safety of the WHO reviewed the safety profiles of the RotaTeq and Rotarix vaccines in 2011 and concluded that both vaccines demonstrated a favorable safety profile. However, the committee acknowledged a potential increased risk of intussusception, with up to a 6-fold increase observed in certain populations within the first 7 days following the initial dose.^[26]^ Despite these findings, the Global Advisory Committee on Vaccine Safety emphasized that the substantial benefits of rotavirus vaccination in preventing severe rotavirus infections far outweigh the associated risks of intussusception.

Several studies have investigated whether vaccines other than the rotavirus vaccine might be associated with intussusception in children. Although the evidence is limited, occasional reports have explored potential links. Early studies hypothesized a connection between the oral polio vaccine and intussusception due to its influence on intestinal lymphoid tissue; however, comprehensive reviews have not established a consistent or significant signal detection.^[27]^ Similarly, measles-containing vaccines have been implicated in rare case reports, but large-scale epidemiological studies have found no conclusive evidence supporting this link.^[28]^ Pentavalent vaccines, which combine protection against multiple diseases, have also been closely monitored for adverse events, including intussusception, yet studies to date have not identified a significant increase in risk. Overall, while the rotavirus vaccine remains the only vaccine consistently associated with a measurable increased risk of intussusception, current evidence does not suggest a meaningful association with other vaccines.^[27,29]^

Unlike previous studies, our research is the first to identify signal detections between intussusception and vaccines other than the rotavirus vaccine. Specifically, our findings suggest that hepatitis B, pneumococcal, and DTP-IPV-Hib vaccines may also contribute to the development of intussusception.

### 4.5. Strengths and limitations

A major strength of this study lies in its use of the WHO’s global pharmacovigilance database, which allowed for an extensive analysis across multiple decades and regions. However, this study has several limitations that warrant consideration. The reliance on a spontaneous reporting system introduces risks of both underreporting in low- and middle-income countries with limited pharmacovigilance infrastructure and overrepresentation of adverse events from regions with robust reporting systems. Additionally, the retrospective design limits control over confounding factors such as preexisting conditions and concomitant medications, while variability in diagnostic criteria and reporting practices across regions may contribute to inconsistencies. Disproportionality analysis, while useful for identifying signal detections, cannot establish causality or account for confounders. Furthermore, the lack of individual-level data, such as vaccine dosage, brand, or genetic predispositions, restricts the ability to fully assess risk factors. These limitations emphasize the need for prospective studies to validate the findings and better understand vaccine-associated intussusception.

## 5. Conclusion

This large-scale analysis emphasizes the safety of vaccines while recognizing the need for vigilance in monitoring rare complications like intussusception, particularly in connection with rotavirus, hepatitis B, pneumococcal, and DTP-IPV-Hib vaccines. The findings underscore the importance of heightened awareness and monitoring, especially in younger populations where the signal detection appears to be elevated. The observed increase in vaccine-associated intussusception since 2010 likely reflects both the widespread introduction of rotavirus vaccines and greater vigilance in clinical practice. While the condition is rare, with a mean TTO of 2.77 days and a low fatality rate of 0.07%, continued pharmacovigilance is essential to ensure early detection and intervention.

Despite the small risk of intussusception, the overall safety profile and benefits of vaccination in preventing severe infectious diseases remain overwhelmingly positive. Future research should focus on elucidating the mechanisms underlying vaccine-associated intussusception and developing strategies to further minimize these risks. Clinicians, patients, and policymakers must collaborate to sustain public confidence in vaccines while reinforcing surveillance systems to promptly address rare adverse events.

## Acknowledgments

The licensing agreement in our database was valid until February 7, 2025 (VigiBase). The perspectives presented herein do not reflect the views of the Uppsala Monitoring Centre or the World Health Organization. We acknowledge that some terms and statements in this manuscript (“association” or “drug-associated”) may be interpreted as implying causality or formal clinical guidance, which was not intended. The dataset is derived from a spontaneous reporting system and disproportionality analysis and therefore does not permit the estimation of incidence or prevalence. This discussion represents the authors’ interpretative perspectives rather than conclusions directly drawn from the database. Therefore, the findings should be approached with caution and are not intended to guide clinical decision-making. The terms “drug-associated intussusception” emerged from a disproportionality analysis, which does not establish a causal relationship. Given that the primary aim of this study was signal detection, careful interpretation of the terminology used is essential.

## Author contributions

**Conceptualization:** Dong Keon Yon.

**Data curation:** Hyesu Jo, Dong Keon Yon.

**Formal analysis:** Tae Hyeong Kim, Hyesu Jo, Dong Keon Yon.

**Funding acquisition:** Dong Keon Yon.

**Investigation:** Tae Hyeong Kim, Hyesu Jo, Dong Keon Yon.

**Methodology:** Hyesu Jo, Dong Keon Yon.

**Project administration:** Tae Hyeong Kim, Hyesu Jo, Dong Keon Yon.

**Resources:** Tae Hyeong Kim, Hyesu Jo, Dong Keon Yon.

**Software:** Hyesu Jo, Dong Keon Yon.

**Supervision:** Hyesu Jo, Dong Keon Yon.

**Validation:** Tae Hyeong Kim, Hyesu Jo, Dong Keon Yon.

**Visualization:** Tae Hyeong Kim, Hyesu Jo, Dong Keon Yon.

**Writing – original draft:** Tae Hyeong Kim, Hyesu Jo, Damiano Pizzol, Lee Smith, Dong Keon Yon.

**Writing – review & editing:** Tae Hyeong Kim, Hyesu Jo, Damiano Pizzol, Lee Smith, Dong Keon Yon.

## Supplementary Material


