# Supplementary material for: Global epidemiology of vaccine-associated intussusception in children and adolescents, 1968 to 2024: An international pharmacovigilance study
Source: Medicine (Baltimore). 2025 Nov 21;104(47):e45878. doi: 10.1097/MD.0000000000045878 (PMC12643591; doi:10.1097/MD.0000000000045878)
Supplement: Supplementary file 1 [file medi-104-e45878-s001.docx]

**Table S1.** Medical Dictionary for Regulatory Activities (MedDRA) preferred terms and classifications for intussusception.

| SOC | HLGT | HLT | PT | LLT | MedDRA code |
| --- | --- | --- | --- | --- | --- |
| Gastrointestinal disorders | Gastrointestinal stenosis and obstruction | Gastrointestinal stenosis and obstruction NEC | Intussusception | Idiopathic intussusception | 10021231 |
| Gastrointestinal disorders | Gastrointestinal stenosis and obstruction | Gastrointestinal stenosis and obstruction NEC | Intussusception | Ileocolic intussusception | 10057087 |
| Gastrointestinal disorders | Gastrointestinal stenosis and obstruction | Gastrointestinal stenosis and obstruction NEC | Intussusception | Intussusception | 10022863 |
| Gastrointestinal disorders | Gastrointestinal stenosis and obstruction | Gastrointestinal stenosis and obstruction NEC | Intussusception | Invagination of colon | 10022874 |
| Gastrointestinal disorders | Gastrointestinal stenosis and obstruction | Gastrointestinal stenosis and obstruction NEC | Intussusception | Invagination of intestine | 10022875 |

HLT, high-level term; HLGT, high-level group term; LLT, lower-level terms; PT, preferred terms; SOC, system organ class.

s
